# Enhancer RNA AL928768.3 from the IGH Locus Regulates MYC Expression and Controls the Proliferation and Chemoresistance of Burkitt Lymphoma Cells with IGH/MYC Translocation

**DOI:** 10.3390/ijms23094624

**Published:** 2022-04-21

**Authors:** Ekaterina Mikhailovna Stasevich, Aksinya Nicolaevna Uvarova, Matvey Mikhailovich Murashko, Elmira Ramilevna Khabusheva, Saveliy Andreevich Sheetikov, Vladimir Sergeyevich Prassolov, Dmitriy Vladimirovich Kuprash, Denis Eriksonovich Demin, Anton Markovich Schwartz

**Affiliations:** 1 Center for Precision Genome Editing and Genetic Technologies for Biomedicine, Engelhardt Institute of Molecular Biology, Russian Academy of Sciences, 119991 Moscow, Russia; stasevich.em@phystech.edu (E.M.S.); uan@eimb.ru (A.N.U.); vr.elmira@gmail.com (E.R.K.); prassolov45@mail.ru (V.S.P.); kuprash@eimb.ru (D.V.K.); shvarts@eimb.ru (A.M.S.); 2 Laboratories for the Transmission of Intracellular Signals in Normal and Pathological Conditions, Engelhardt Institute of Molecular Biology, Russian Academy of Sciences, 119991 Moscow, Russia; murashko.mm@phystech.edu; 3 Department of Molecular and Biological Physics, Moscow Institute of Physics and Technology, 141701 Moscow, Russia; 4 Department of Cancer Cell Biology, Engelhardt Institute of Molecular Biology, Russian Academy of Sciences, Vavilov Street 32, 119991 Moscow, Russia; 5 National Research Center for Hematology, 125167 Moscow, Russia; sheetikov.s@blood.ru

**Keywords:** eRNA, IGH/MYC, translocation, oncogene, Burkitt lymphoma

## Abstract

Chromosomal rearrangements leading to the relocation of proto-oncogenes into transcription-active regions are found in various types of tumors. In particular, the transfer of proto-oncogenes to the locus of heavy chains of immunoglobulins (IGH) is frequently observed in B-lymphomas. The increased expression of the MYC proto-oncogene due to IGH/MYC translocation is detected in approximately 85% of Burkitt lymphoma cases. The regulatory mechanisms affecting the oncogenes upon translocation include non-coding enhancer RNAs (eRNAs). We conducted a search for the eRNAs that may affect MYC transcription in the case of IGH/MYC translocation in Burkitt lymphoma, looking for potentially oncogenic eRNAs located at the IGH locus and predominantly expressed in B cells. Overexpression and knockdown of our primary candidate eRNA AL928768.3 led to the corresponding changes in the expression of MYC proto-oncogene in Burkitt lymphoma cells. Furthermore, we demonstrated that AL928768.3 knockdown decreased lymphoma cell proliferation and resistance to chemotherapy. Significant effects were observed only in cell lines bearing IGH/MYC abnormality but not in B-cell lines without this translocation nor primary B-cells. Our results indicate that AL928768.3 plays an important role in the development of Burkitt’s lymphoma and suggest it and similar, yet undiscovered eRNAs as potential tissue-specific targets for cancer treatment.

## 1. Introduction

B and T cells are extremely susceptible to genomic rearrangements, which is probably related to active genomic recombination during the diversification of specific antigen receptors and antibodies. As a result, translocations are observed frequently in various blood cancers [1]. The increased expression of proto-oncogenes may result from translocation due to fusion with another gene or because of the influence of new cis-regulatory elements. The genes c-MYC, NOTCH1, TLX1, LMO1 and LMO2 are commonly moved under the control of T-cell receptor (TCR) regulatory elements in T-cell acute lymphoblastic leukemia (T-ALL) [2]. Likewise, in B-cell malignancies, proto-oncogenes are often translocated to the locus of the immunoglobulin heavy chain (IGH) [3], such as MYC in Burkitt lymphoma (IGH/MYC), CCND1 in mantle cell lymphoma, BCL-6 in diffuse large B-cell lymphoma (DLBCL) and BCL-2 in follicular lymphoma [4].

Burkitt lymphoma is a highly malignant type of blood cancer. Translocation of the MYC gene (8q24) to the immunoglobulin heavy chain locus (14q32) that leads to its overexpression is detected in approximately 85% of Burkitt lymphoma cases. In rarer cases, MYC is translocated to the immunoglobulin kappa locus or the immunoglobulin lambda locus [5,6]. Burkitt lymphoma is most prevalent in some regions of Africa, where the disease is associated with Epstein–Barr virus (EBV) infection. It was proposed that EBV may participate in Burkitt’s lymphoma development in the case of MYC translocation [7]. Indeed, increased MYC expression in healthy cells leads to p53-dependent or p53-independent apoptosis that can be inhibited by the viral EBNA-1 protein and EBV-encoded RNAs [8]. These mechanisms explain the close connection between EBV infection, MYC translocations and the development of a lymphoproliferative process.

MYC is an ordinary proto-oncogene; its expression changes in 70% of human tumors [9]. Abnormal MYC expression can lead to genomic instability, uncontrolled cell growth and escape from the immune response [10]. A number of studies demonstrate that suppression of MYC expression leads to reduced cell proliferation in cancer cell lines [11,12]. Drug design for direct suppression of MYC activity remains challenging because c-MYC protein has no apparent “pockets” for small molecule binding. C-MYC is located primarily in the nucleus where it is inaccessible to antibodies [13]. Among other things, there are numerous possible side effects of suppressing c-MYC directly, because in healthy cells it is an important transcription factor involved in cell division, differentiation, maintenance of stemness and cellular metabolism [14]. Direct control of MYC expression in tumor cells without exuberant stress on healthy tissues remains an unsolved task. We suggest that in tumor cells with IGH/MYC translocation, this task may be tackled by affecting the activity of IGH locus enhancers.

Several studies have shown that suppression of the activity of certain enhancers can be achieved by suppressing the expression of the corresponding enhancer RNAs (eRNAs) [15,16,17], a subgroup of long non-coding RNAs (lncRNAs) transcribed from the enhancer regions. Enhancers control tissue-specific gene expression, thus eRNAs’ expression is also unique to each cell type. eRNAs are frequently localized in the nucleus and relatively unstable compared to mRNA. Several reports confirm that eRNAs have many important cellular functions, such as chromatin modification and regulation of transcription [18,19]. Some researchers suggest that eRNAs can be cancer biomarkers, for example, for head and neck squamous cell carcinoma and lung squamous cell carcinoma [20,21].

There are several mechanisms by which eRNA can regulate gene expression. eRNA may stabilize the enhancer-promoter loop, interact with transcription factors, promote histone modification or facilitate the transition of RNA polymerase II (Pol II) at target gene promoters [22]. eRNAs can stimulate the interaction of the RNAPII with promoters through deactivation of the negative elongation factor (NELF) complex [23] and activation of the positive transcription elongation factor b (P-TEFb) complex [24]. A number of studies confirm the ability of eRNA to stabilize the enhancer-promoter loop. For instance, it has been shown that the regulation of estrogen-upregulated coding genes proceeds through stabilizing the enhancer-promoter loops with eRNA [25]. One formation mechanism of such loops that has been demonstrated includes stabilization of these chromosomal structures via interaction with the cohesin complex [15]. A number of studies showed the ability of eRNA to increase enhancer activity by attracting transcription factors and epigenetic modifying enzymes such as BRD4 [26], hnRNPL that promotes H3K36me3 histone modification [27] and CBP/p300 complex that produces the H3K27ac chromatin-activating modification [28].

eRNAs can be divided into two major classes depending on whether their enhancers are transcribed in both directions. The 1D-eRNAs which result from a unidirectional transcription of the enhancer are typically long (>150 nt), polyadenylated and spliced. 1D-eRNAs are generally more stable and may be involved in the regulation of distant gene expression. The eRNAs of the second type called 2D-eRNA are expressed from both strands and are usually shorter than 1D-eRNAs. 2D-eRNAs are not spliced, lack a poly-A tail and usually function at the same locus [29].

Thereby, eRNAs appear to be important participants in the regulation of gene expression. The search for eRNAs involved in the regulation of proto-oncogenes uncovered new mechanisms of carcinogenesis of tumors carrying chromosomal abnormalities. Such eRNAs could be unique targets for the selective suppression of proto-oncogene expression in tumor cells. In this study, we performed a search and functional analysis of eRNAs in the IGH locus expressed in Burkitt lymphoma cells and capable of stimulating the expression of the MYC gene.

## 2. Results

### 2.1. The Search for Potential eRNAs from the IGH Locus

**Figure 1:**
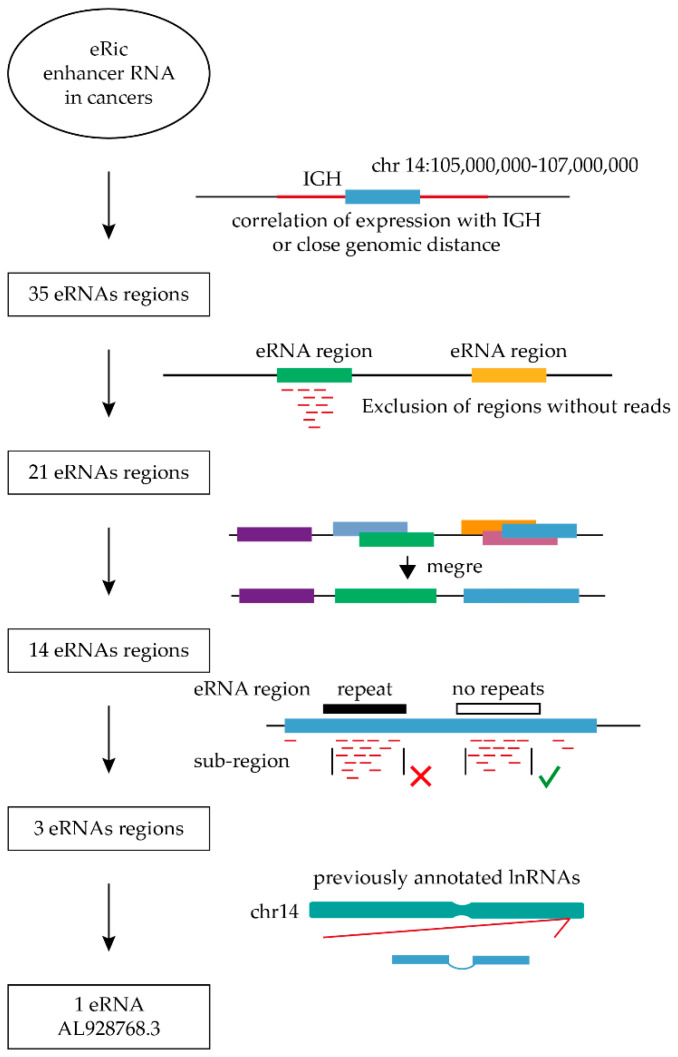
The scheme of the bioinformatics search. The first diagram shows a schematic representation of the IGH locus. The second illustration shows a schematic representation of the alignment and the third diagram presents the regions before and after the merge. The fourth diagram illustrates the identification of sub-regions densely covered with reads followed by the exclusion of sub-regions overlapping with the genomic repeats. Only one region of three has previously annotated lncRNA. The fifth diagram shows schematically chromosome 14 and the region containing RNA.

We used the eRic database [30] to obtain 35 IGH-related eRNA regions (Figure 1).

Upon discarding the regions with no RNA-seq reads from cell lines with IGH/MYC translocation and merging the overlapping regions, the number of regions was reduced to 14. For every merged eRNA region, we outlined the sub-regions densely covered by RNA-seq reads (Table S1). Since our search was focused on finding a potential target for the treatment of Burkitt lymphoma, it was important to find a unique sequence to ensure specificity. Therefore, we eliminated the sub-regions coinciding with repetitive elements which left 3 eRNA regions with the reads aligned to a unique sequence. Among these three remaining eRNA regions, we found one region containing the previously annotated lncRNA AL928768.3. This eRNA was investigated further.

### 2.2. Analysis of AL928768.3 eRNA Expression in Human Blood Cells

**Figure 2:**
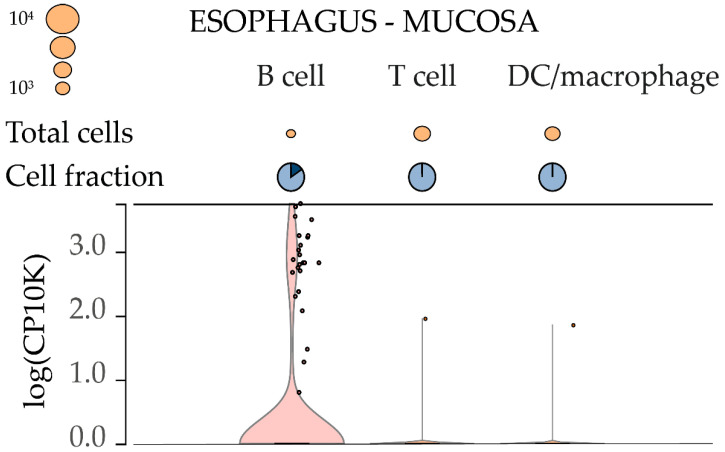
Analysis of AL928768.3 expression using available single-nucleus RNA sequencing data. In cell types other than B-lymphocytes, AL928768.3 expression was detected only in a few individual cells.

At the next stage of the study, we evaluated the specificity of eRNA expression in B cells and the relationship of its level with the transcriptional activity of the IGH locus. An analysis of available data on single nucleus RNA sequencing (snRNA-seq) from the Genotype-Tissue Expression (GTEx) portal [31] showed that the nuclear expression of AL928768.3 is specific to B-lymphocytes (Figure 2).

**Figure 3:**
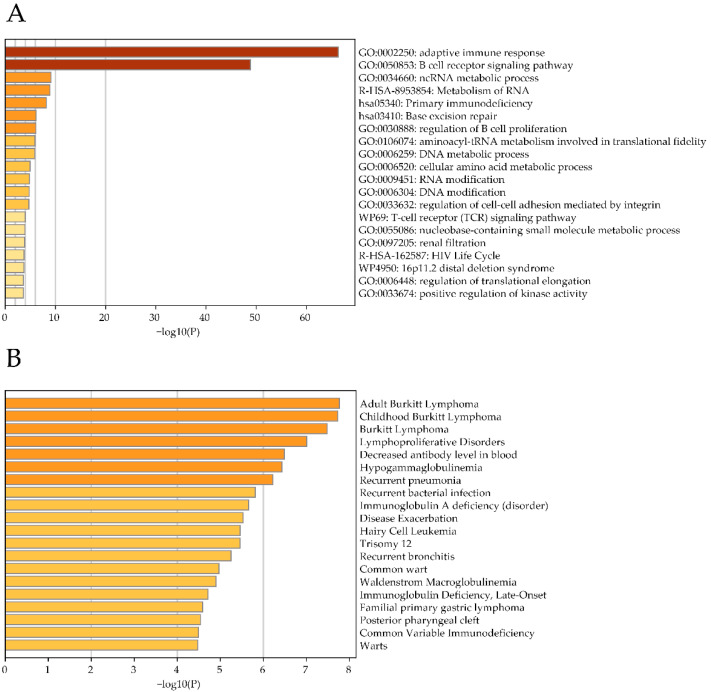
Analysis of AL928768.3 eRNAs expression in human blood cells. Clustering of genes co-expressed with eRNAs of AL928768.3 in blood cells by functional ontologies (**A**) and by disease association (**B**).

To assess the conditions under which AL928768.3 is expressed, we performed an analysis of the transcriptomes of 755 human leukocyte samples from the GTEx database. It showed that the level of this RNA was elevated in samples with high expression levels of various types of immunoglobulins as well as with the genes associated with B-lymphocyte activation (Figure 3A, Table S2). This is consistent with our hypothesis that AL928768.3 is perhaps associated with the regulation of the IGH locus. Additionally, using the DisGeNET database of disease genomics [32], we demonstrated that elevated levels of eRNAs AL928768.3 correlated with those genes particularly characteristic of individuals with Burkitt lymphoma. Of note, the correlation was observed for both the adult and childhood variants of this disease (Figure 3B). In addition, according to GTEx data, a high level of AL928768.3 was also observed in the B-lymphocytes infected with EBV (Figure S1). As mentioned, most cases of Burkitt lymphoma are associated with EBV infection [7].

### 2.3. Effect of eRNA Knockdown on MYC Expression, Cell Proliferation and Chemoresistance

**Figure 4:**
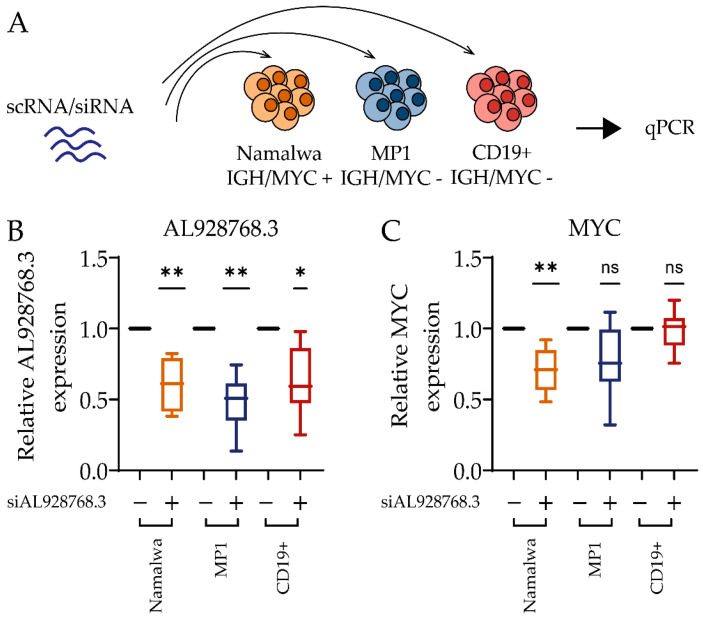
The siRNA-mediated knockdown of AL928768.3. (**A**) The scheme of the experiment. Cells were electroporated with siRNA targetingAL928768.3 (+) or scRNA as a control (−). Expression data were normalized on values in control samples. The diagrams demonstrate relative AL928768.3 (**B**) and MYC (**C**) expression. * *p*-value less than 0.05; ** *p*-value less than 0.01, ns—no significant difference (Wilcoxon test). The number of independent experiments: Namalwa (n = 8); MP1 (n = 8); CD19+ primary B cell (n = 6).

In order to verify the hypothesis that AL928768.3 influences the expression of MYC in Burkitt lymphoma cells, we used Namalwa cell line, which is a Burkitt lymphoma carrying an IGH/MYC translocation. MP1, a B-lymphoblastoid cell line without IGH/MYC, and CD19+ primary B cells were used as controls (Figure 4A). The knockdown of AL928768.3 by RNA interference resulted in an approximately two-fold decrease in the median eRNA expression of all cell types (Figure 4B). However, a significant effect of AL928768.3 knockdown on MYC proto-oncogene expression was only observed in Namalwa cells, not in MP1 and CD19+ cells (Figure 4B).

**Figure 5:**
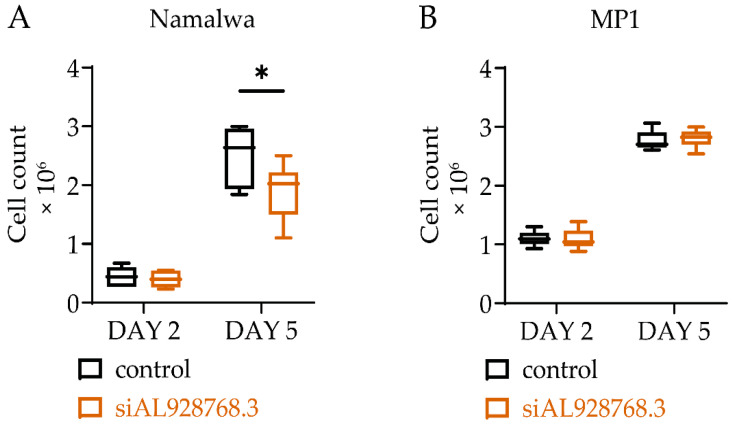
Cell proliferation assay in Namalwa (**A**) and MP1 (**B**) cell lines with the knockdown of AL928768.3. Measurements were taken on the second and fifth days. ScRNA was used as a control. * *p*-value less than 0.05 (Wilcoxon test). The results of six independent experiments are shown.

For a diverse range of B-cell lymphomas, a reduced level of MYC expression has been associated with patient survival [33]. This could be attributed to the tight relationship between c-MYC transcription factor and cell proliferation. Hence, our next step was to study the possibility of selective suppression of the proliferation of cells with an IGH/MYC translocation by the knockdown of eRNA AL928768.3 (Figure 5). On the 5th day of the experiment, the number of Namalwa cells with the AL928768.3 knockdown was significantly lower than the control with scrambled siRNA (scRNA) while no alteration in the MP1 cell growth rate was observed. Therefore, a two-fold decrease in the AL928768.3 eRNA level led to significant suppression of Burkitt lymphoma cell proliferation in our experimental model.

**Figure 6:**
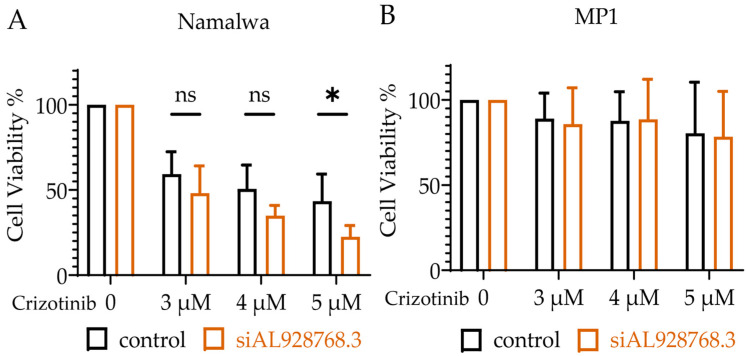
Cell viability under Crizotinib with the knockdown of AL928768.3. Number of viable Namalwa (n = 4) (**A**) and MP1 (n = 4) (**B**) cells after treatment with 3 μM, 4 μM and 5 μM of Crizotinib. Measurements were taken on day 5th. Normalization was performed on the same sample without the drug. ScRNA was used as a control. * *p*-value less than 0.05, ns—no significant difference (Student’s *t*-test). The results of 4 independent experiments are shown.

We next explored the effect of the expression of AL928768.3 on chemoresistance. Several articles have reported the correlation between increased MYC expression and resistance to Crizotinib in lung cancer [34,35] and lymphoma cells [36]. The knockdown of AL928768.3 resulted in significantly increased sensitivity to Crizotinib at the concentration of 5 μM (Figure 6A). The resistance of MP1 cells remained unchanged with a decreased expression of AL928768.3 at the same drug concentrations (Figure 6B). An analysis of the fraction of apoptotic cells showed no significant difference, regardless of the AL928768.3 expression (Figure S2). Thus, the observed lower number of cells in the samples with reduced levels of RNA AL928768.3 is more likely due to its effect on the rate of cell division.

### 2.4. Effect of eRNA Overexpression on MYC Expression

**Figure 7:**
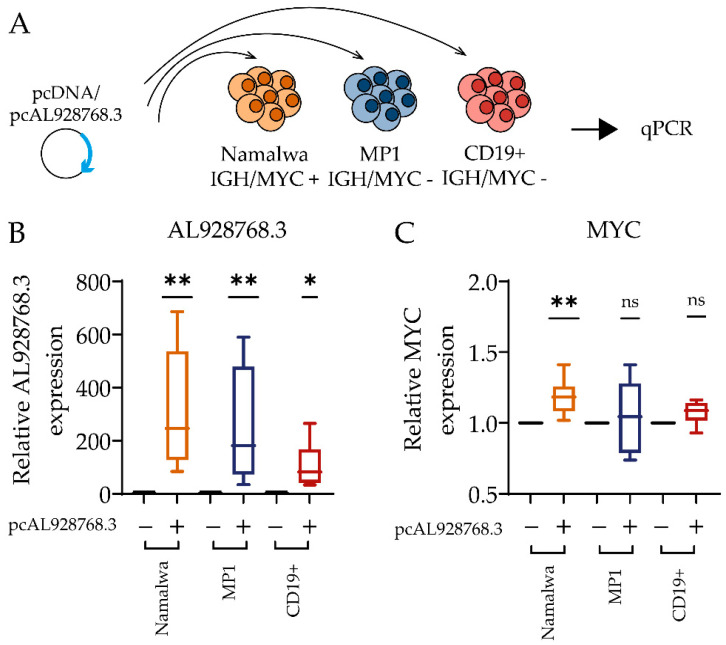
Overexpression of eRNA AL928768.3. (**A**) The scheme of the experiment. Namalwa (n = 8), MP1 (n = 8) cell lines and CD19+ primary B cell (n = 6) were electroporated with plasmid for AL928768.3 expression. The empty vector (pcDNA) was used as a control. Expression data were normalized on the values of the control samples. The figure demonstrates the relative AL928768.3 (**B**) and MYC (**C**) expression in Namalwa, MP1 cell lines and CD19+ primary B cells. * *p*-value less than 0.05; ** less than 0.01, ns—no significant difference (Wilcoxon test).

The result of the previous experiment implies that AL928768.3 knockdown affects MYC gene expression only if these genes are located in proximity. As mentioned in the introduction, an eRNA can have a direct stimulatory effect on a promoter. If this is the case, the effect of AL928768.3 on the distant MYC promoter should depend on the eRNA expression level and its distribution in the nucleus. To test this hypothesis, we performed an overexpression experiment (Figure 7A). Expression of AL928768.3 from exogenous DNA resulted in an increase in the median expression by more than two orders of magnitude (Figure 7B). In Namalwa cells, an overexpression of AL928768.3 resulted in a small but significant change in MYC expression, whereas no effect was observed in MP1 or in primary B cells (Figure 7C). Thus, AL928768.3 is unable to directly affect the MYC promoter and has an effect on MYC expression only if the gene is located at the IGH locus.

## 3. Discussion

Enhancers are important transcriptional regions that can control the tissue-specific expression of various genes, including oncogenes. The number of publications mentioning non-coding RNAs transcribed from enhancer regions (enhancer RNAs) continues to grow, however, it remains a poorly explored area. Since the discovery of eRNAs, there have been several hypotheses about their function. One suggestion is that eRNAs are transcriptional noise and have no specific function. Another possibility is that it is the transcription process rather than the eRNA itself that is essential to the enhancer operation. The third idea, gaining more and more evidence in various studies, suggests that eRNAs themselves can influence gene expression [18]. It has also been shown that eRNAs can work both in trans and in cis [19].

In our study, we confirm that eRNAs may be involved in new regulatory interactions between enhancers and promoters resulting from chromosomal rearrangements. Suppression of the AL928768.3 eRNA expression allowed selective downregulation of MYC gene expression and the inhibition of cell growth in Burkitt lymphoma cells, but not in B cells without IGH/MYC abnormality. According to the previously described classification, AL928768.3 belongs to the class 1D eRNA since it appears to be transcribed unidirectionally, is more than 150nt long and is spliced.

MYC is an important transcription factor in healthy cells that are involved in cell division, differentiation, maintenance of stemness, cellular metabolism and other functions. MYC also has the characteristics of a proto-oncogene and is implicated in the formation of drug resistance in tumor cells [10]. Selective suppression of MYC expression could be a potential component of lymphoma therapy, including the treatment of drug-resistant tumors. Our results indicate that this area deserves further research. It is especially interesting to examine the effect of AL928768.3 eRNA in the regulation of other oncogenes (CCND1, BCL-6) that are translocated into the IGH locus as a result of chromosomal rearrangements in B-cell lymphomas. Further investigation of AL928768.3, as well as the search for new eRNAs, may bring about a better understanding of the contribution of eRNAs to the development of cancer as well as other diseases. For example, eRNA AL928768.3 levels have been shown to be elevated in patients with rheumatoid arthritis [37], but whether this eRNA plays a role in the pathogenic inflammation is unknown.

The search for new eRNAs and approaches to their regulation might be a complicated issue. A weak correlation between activity and eRNA levels has been shown for many enhancers [38]. Moreover, some strong enhancers are barely transcribed [39]. In addition, some eRNAs can affect distant genes [15], which might complicate the identification of such genes. It was also found that the activity and direction of the transcription of particular enhancers depend on the individual genetic features [40].

The influence of the eRNA AL928768.3 on its own locus can be demonstrated by the effects of the AL928768.3 knockdown on the expression of nearby genes. In the cell lines under study, the AL928768.3 results in a decrease in the expression level of the IGHA1 gene and a smaller, yet detectable effect on the more distant IGHG1 gene (Figure S3). This may indicate that the effects of this eRNA depend on the mutual location of the corresponding enhancer of the IGH locus and the translocated MYC gene.

Suppression of eRNA expression is achieved mainly through the use of the siRNA or dCas9/KRAB system [15,16,17]. There is active development of siRNA-based medical drugs for many diseases, including cancer therapy [41], as well as the development of approaches using CRISPRi/dCas9 technology [42]. Medical preparations based on RNA and viral vectors are already used in medical practice both for vaccination and for the treatment of genetic diseases [43,44,45,46]. The combination of these advances with the characterization of eRNAs that selectively control the activity of oncogenes in tumor cells could lead to the development of new anticancer therapies.

## 4. Materials and Methods

### 4.1. Search for eRNAs for Functional Analysis

In order to find the eRNAs that potentially can influence MYC expression in case of its translocation to IGH locus (chr14:105,000,000–107,000,000 in hg38), we used the eRic database (enhancer RNA in cancers) [30] that represents eRNA regions as segments of 6000 bp (±3000 bp from the enhancer ChIP-seq peak). In addition to the eRic database entries located in the IGH locus, candidate regions also included those correlated in expression with IGH in human cancers. We then mapped RNA-seq reads from B-cell lines harboring IGH/MYC to the candidate eRNA regions. Data for the analysis were extracted from the NCBI Sequence Read Archive (SRA) (https://www.ncbi.nlm.nih.gov/sra accessed on 20 May 2020). Five cell lines with IGH/MYC were used: RAJI (SRR3956932 and DRR008652), Daudi (DRR062881), Akata (DRR057224), BCBL-1 (SRR7685960), NAMALWA (SRR8311059). One mismatch in a read was allowed, regions with zero aligned reads were eliminated and overlapping regions covered by reads were combined. Following this, for each eRNA segment, we selected sub-regions densely covered with reads as the most probable area of a transcript. These sub-regions were filtered for the absence of genome repeats (short interspersed nuclear elements (SINE), long interspersed nuclear elements (LINE), long terminal repeat elements (LTR) or other DNA repeat elements (DNA)) (Table S1). For the remaining subregions, we carried out a search for previously annotated long noncoding RNAs using the ENSEMBL database.

### 4.2. Cell Culture and Transfection

B-lymphoblastoid cell line MP1 [47] and Burkitt lymphoma cell line Namalwa (kindly provided by Dr. Edward A. Clark, University of Washington, Washington, DC, USA) were maintained in an RPMI 1640 medium (PanEco, Moscow, Russia). The culture medium was supplemented with 10% FBS (Corning, NY, USA), 2 mM L-glutamine (PanEco, Moscow, Russia), 100 U/mL penicillium и 100 mg/mL streptomycin (PanEco, Moscow, Russia), 1× non-essential amino acids (GIBCO, Kwartsweg, The Netherlands), 10 mM HEPES (GIBCO, Kwartsweg, The Netherlands) and 1 mM sodium pyruvate (PanEco, Moscow, Russia). The CD19+ cells were isolated from the peripheral blood mononuclear cells of healthy donors using the human CD19 MACS Cell Isolation Kit (Miltenyi Biotec, Bergisch Gladbach, Germany). Cell activation was performed by adding phorbol myristate acetate (PMA) (Sigma-Aldrich, Burlington, MA, USA) at a concentration of 50 ng/mL to the culture medium. All donors signed the informed consent form approved by the National Research Center for Hematology Ethics Committee before enrollment.

For overexpression experiments, the AL928768.3 DNA was amplified from total Namalwa cDNA and cloned in pcDNA3.1 Hygro+ mammalian expression vector (Invitrogen, Waltham, MA, USA). The empty vector pcDNA3.1Hygro+ was used as a control and pEGFP-N3 (Clontech, Mountain View, CA, USA) was used to evaluate the effectiveness of transfection. AL928768.3 expression was suppressed using sequence-specific siRNA (Table 1). The control scRNA was designed by siRNA Wizard Tool (InvivoGen, San Diego, CA, USA). Transfection was performed using electroporation with the Neon Transfection System (Life Technologies, Kwartsweg, The Netherlands) by one 30-ms and 1300 V impulses for the MP1 cell line and two 20-ms and 1350 V impulses for Namalwa in 100 μL tips designed for this instrument. Five million cells were transfected with 5 μg of plasmid or 500 pmol siRNA. The transfection efficiencies under these conditions were approximately 40% for MP1 cells and 15% for Namalwa (Figure S4).

### 4.3. RNA Isolation and Quantitative Real-Time Polymerase Chain Reaction (qPCR) Analysis

Afterward, the electroporation cells were cultured in a complete medium for 24 h and lysed in TRIzol reagent for total RNA isolation. RNA quantity and quality were estimated with a spectrophotometer (NanoDrop). The total RNA was reverse-transcribed into first-strand cDNA using an MMLV RT kit (Evrogen, Moscow, Russia) and 1:1 mixed Oligo (dT) and random primers. Real-time PCR analysis was performed using the CFX96 Touch Real-Time PCR Detection System (Bio-Rad Laboratories, Hercules, CA, USA) and qPCR mix-HS SYBR (Evrogen, Moscow, Russia). GAPDH was used as a reference gene. The sequences of the oligonucleotide primers are presented in Table 1.

### 4.4. Cell Proliferation Assay and Chemoresistance

Namalwa and MP1 cell lines were transfected with AL928768.3 sequence-specific siRNA and scRNA and were cultured in a complete medium for 24 h. Transfected cells were seeded at a density of 2 × 10^5^ cells/mL in triplicate, in 24-well plates. The number of cells was measured using the Countess II FL Automated Cell Counter (Thermo Fisher Scientific, Waltham, MA, USA) on the 2nd and 5th days after seeding.

Similarly, for monitoring cell viability under Crizotinib (Sigma-Aldrich (USA) PZ0191), Namalwa and MP1 cell lines were seeded at a density of 2 × 10^5^ cells/mL in triplicate, in 96-well plates with a volume of 100 μL/well. Crizotinib was added at concentrations of 3 μM, 4 μM and 5 μM. On the fifth day after treatment, cells were incubated with MTS reagent (Abcam, Cambridge, UK) for 2 h and analyzed by Microplate Photometer (Thermo Fisher Scientific, Waltham, MA, USA). The percent of apoptotic cells was assessed by the BD LSRFortessa Flow Cytometer (BD Biosciences, Franklin Lakes, NJ, USA). Cells were stained with Annexin V-FITC (Molecular Probes, ThermoFisher, Paisley, Renfrewshire, Scotland, UK) and propidium iodide (Sigma Aldrich, Saint Louis, MO, USA), as described previously [48].

### 4.5. Correlational Analysis

To find the genes co-expressed with AL928768.3 in normal whole blood, the Spearman correlation coefficient was calculated using GTEx RNAseq data. The data used for the analyses were obtained from dbGaP accession number phs000424.v8.p2. For gene group enrichment analysis using the Metascape service [49], the top 500 genes with the lowest *p*-values that correlated with AL928768.3 expression were used.

### 4.6. Statistical Analysis

GraphPad Prism 9 software was used for statistical analysis of the obtained data. *p*-values less than 0.05 were considered statistically significant.

## Figures and Tables

**Table 1:** Oligonucleotide sequences for sequence-specific siRNA and primers for qPCR.

Oligonucleotides	Sequence (5′–3′)
siRNA AL928768.3 F	UCUGCAACACAGCAAGAGCdTdT
siRNA AL928768.3 R	GCUCUUGCUGUGUUGCAGAdTdT
scRNA AL928768.3 F	GGAGAAUAGCCCCAACACUdTdT
scRNA AL928768.3 R	AGUGUUGGGGCUAUUCUCCdTdT
AL928768.3_qPCR F	CACAGGGAGGAAGTGTGGAG
AL928768.3_qPCR R	GGGCCACTTTATTGCACCTG
c-Myc_qPCR F	AGCCCCGAGCCCCTGGTG
c-Myc_qPCR R	GGCGCTGCGTAGTTGTGCTGATGT
GAPDH_qPCR F	CAAGGTCATCCATGACAACTTTG
GAPDH_qPCR R	GGCCATCCACAGTCTTCTGG
IGHA1 qPCR F	ACATGCCACGTGAAGCACT
IGHA1 qPCR R	GCACGTGAGGTTCGCTTCT
IGHG1 qPCR F	CAGGACTCTACTCCCTCAGCA
IGHG1 qPCR R	ATGAGGGTGTCCTTGGGTTT

## Data Availability

Not applicable.

## Supplementary Materials

The following supporting information can be downloaded at: https://www.mdpi.com/article/10.3390/ijms23094624/s1.
