# Supplementary material for: Enhancer RNA AL928768.3 from the IGH Locus Regulates MYC Expression and Controls the Proliferation and Chemoresistance of Burkitt Lymphoma Cells with IGH/MYC Translocation
Source: Int J Mol Sci. 2022 Apr 21;23(9):4624. doi: 10.3390/ijms23094624 (PMC9103539; doi:10.3390/ijms23094624)

Figure S1. Analysis of AL928768.3 expression in different tissues using bulk RNA-seq.

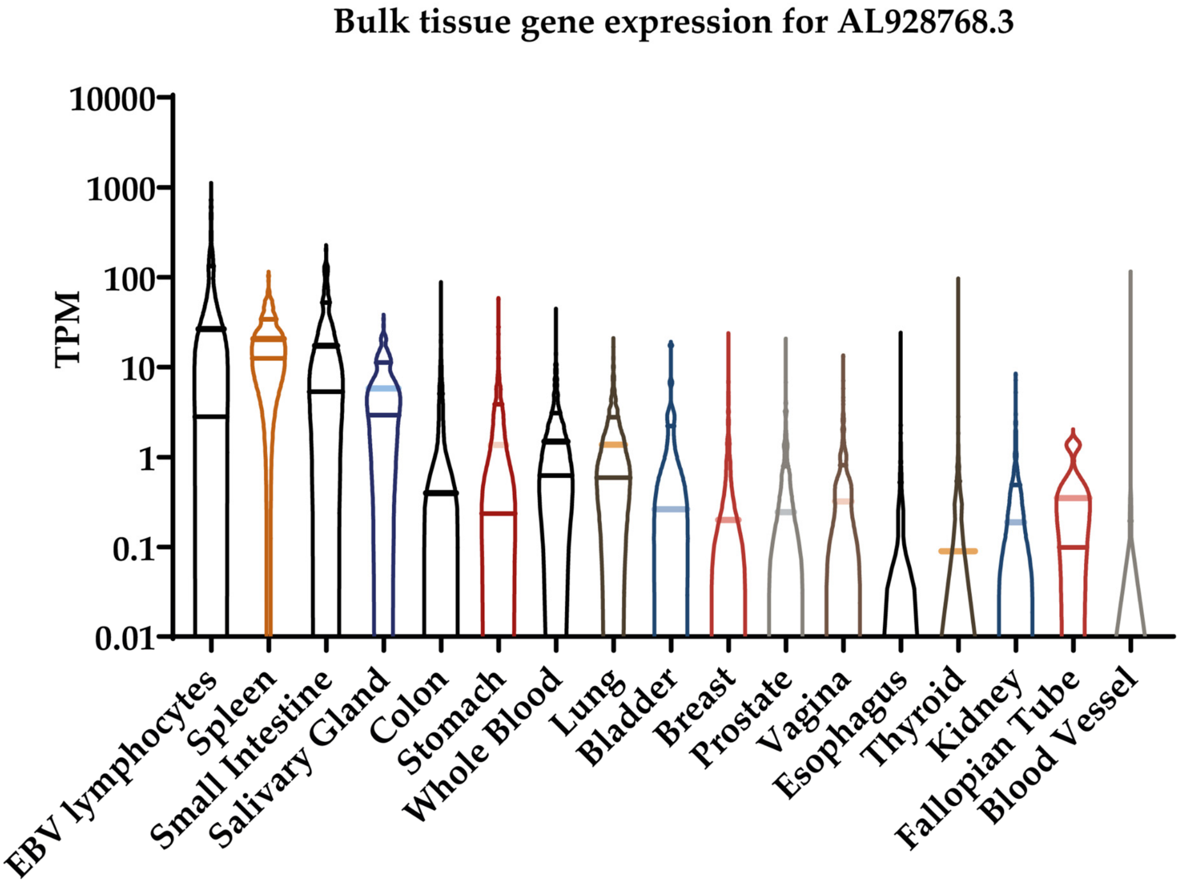

Figure S2. Proportions of alive, dead and apoptotic cells during crizatinib treatment and knockdown of AL928768.3 determined using FACS. Control sample and sample with crizatinib were transfected with scramble siRNA. A, B, C – MP1 cell line, D, E, F – Namalwa cell line.

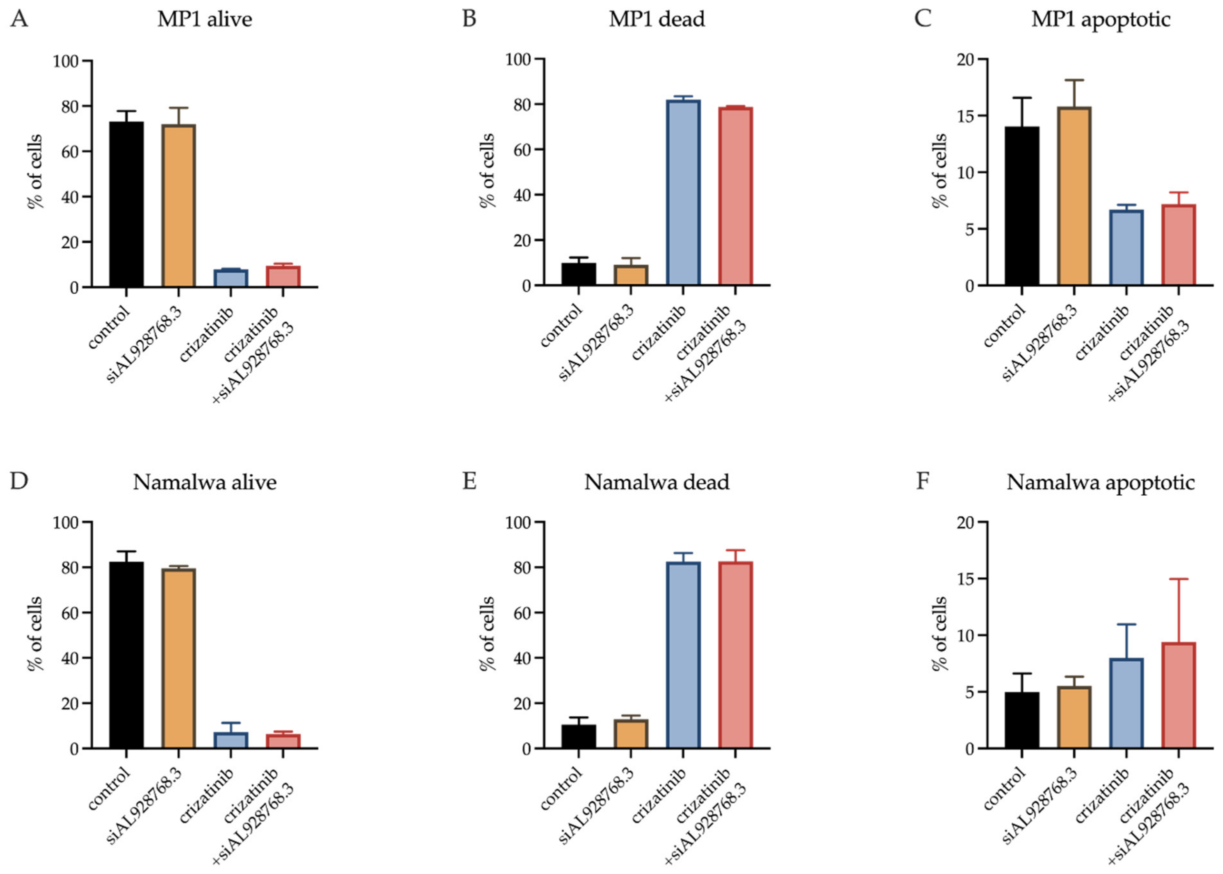

Figure S3. The impact of AL928768.3 knockdown on expression of neighboring genes IGHA1 and IGHG1. Expression levels were measured using quantitative PCR. The effects of the knockdown were measured in A. MP1 and B. Namalwa cell lines. C. Location of genes in the genome.

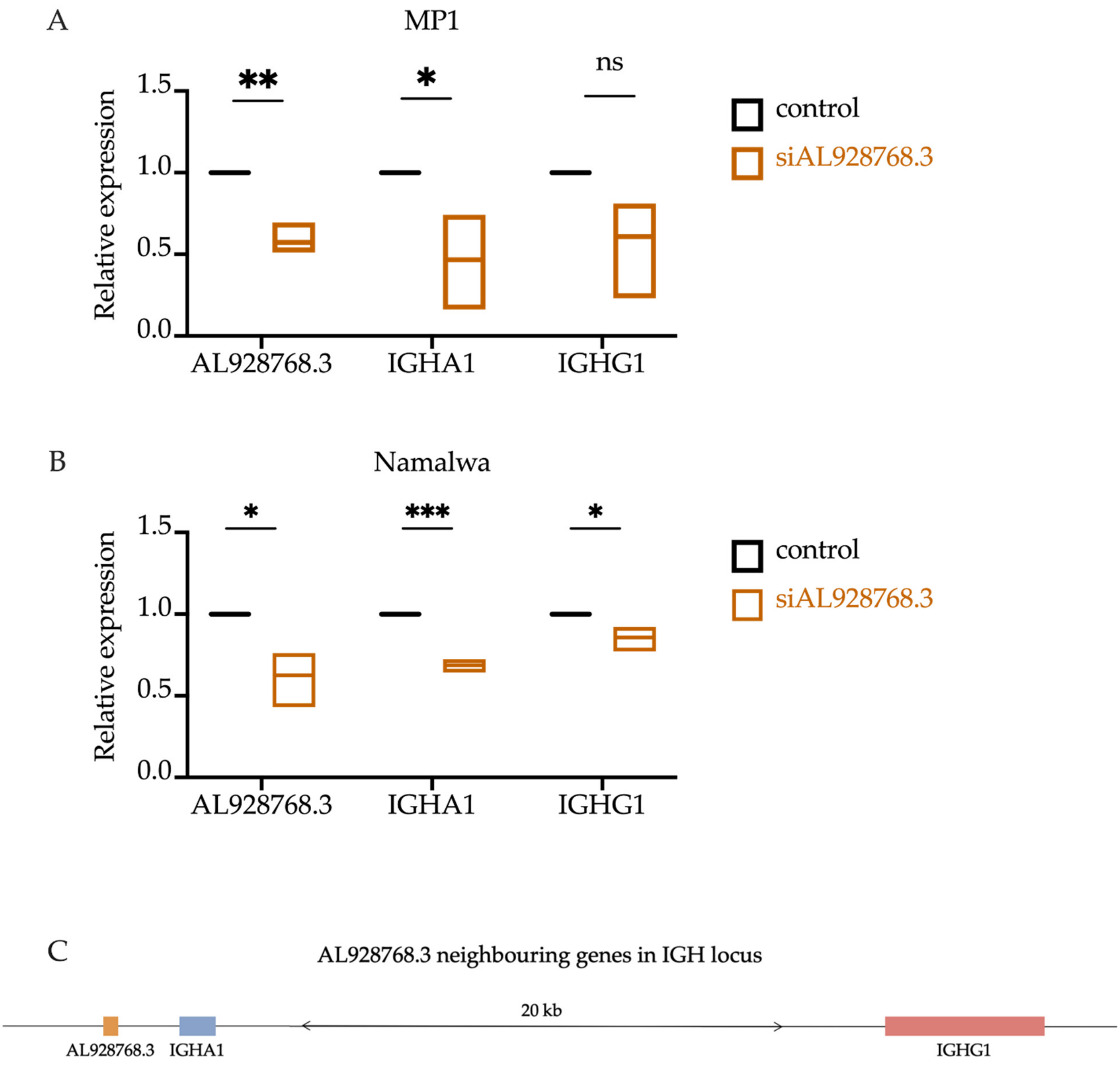

Figure S4. The proportions of GFP expressing cells during transfection. Populations of GFP positive MP-1 and Namalwa cells were determined using FACS.

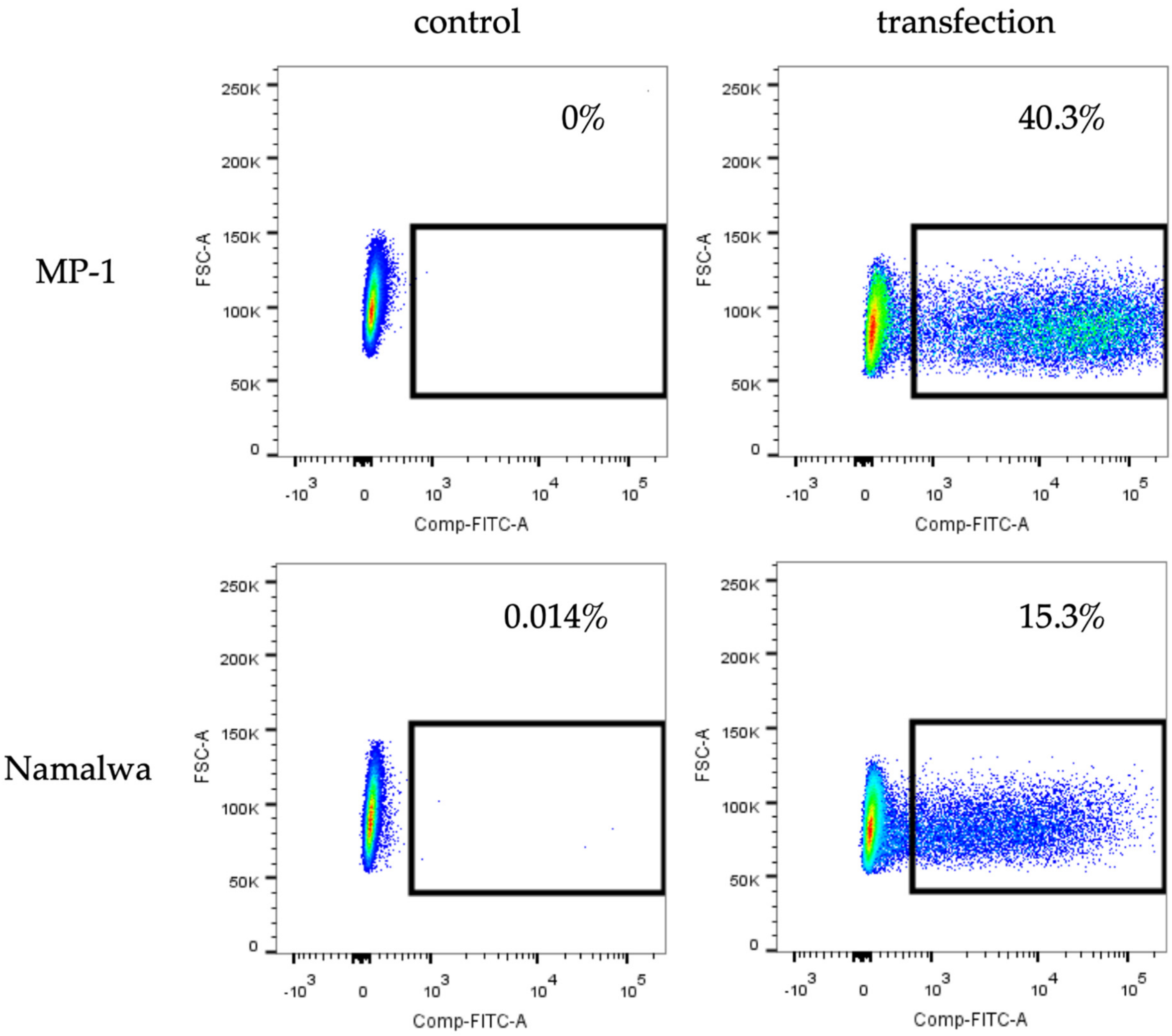

Supplement: Supplementary file 1 [file ijms-23-04624-s001.zip › Figures S1¿CS4.pdf]
