# Supplementary material for: Enhancer RNA AL928768.3 from the IGH Locus Regulates MYC Expression and Controls the Proliferation and Chemoresistance of Burkitt Lymphoma Cells with IGH/MYC Translocation
Source: Int J Mol Sci. 2022 Apr 21;23(9):4624. doi: 10.3390/ijms23094624 (PMC9103539; doi:10.3390/ijms23094624)
Supplement: Supplementary file 1 [file ijms-23-04624-s001.zip › Table S1.pdf]

Table S1. Schematic illustration of the regions, alignment reads and genome repeats. The illustration shows alignment of Raji cell line RNA-Seq reads (SRX1981036) from Sequence Read Archive Nucleotide BLAST (NSBI). The positions of genome repeats were obtained from UCSC Genome Browser on Human (hg38) Assembly. The three areas that were selected are highlighted in green.

#	Coordinates of region (hg 38)	Alignment of reads and repeats position
1	11:68378000-68384000	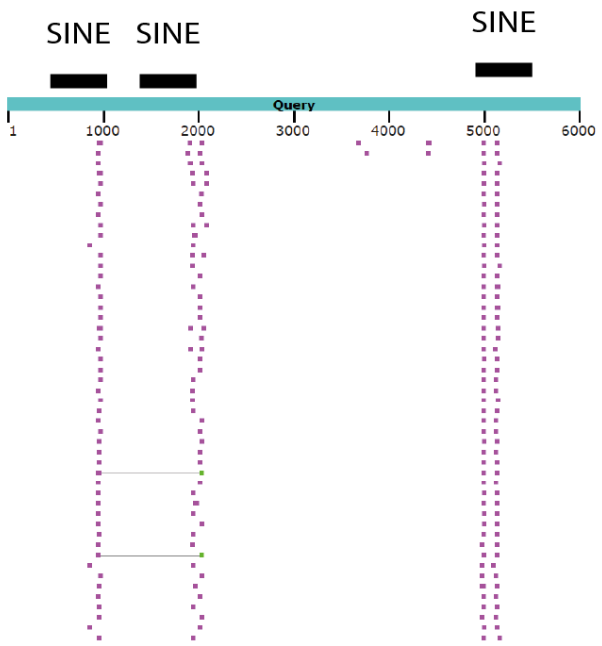
2	11:68576671-68582671	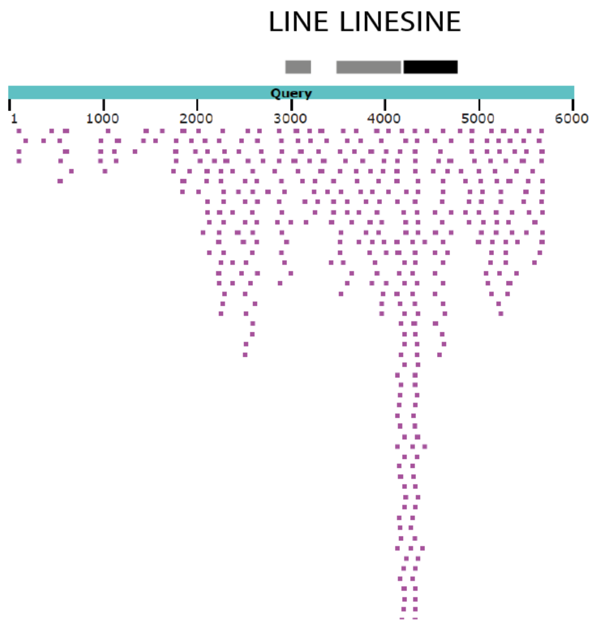

3	11:68947446-68953446	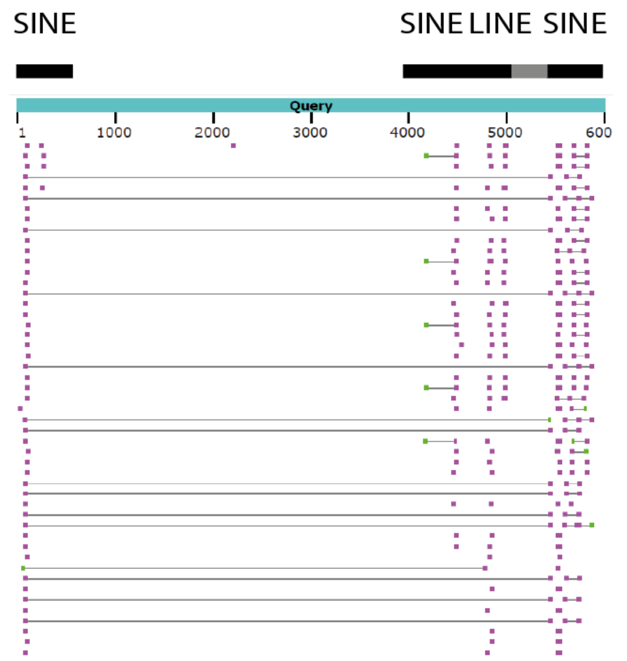
4	11:69379400-69391400	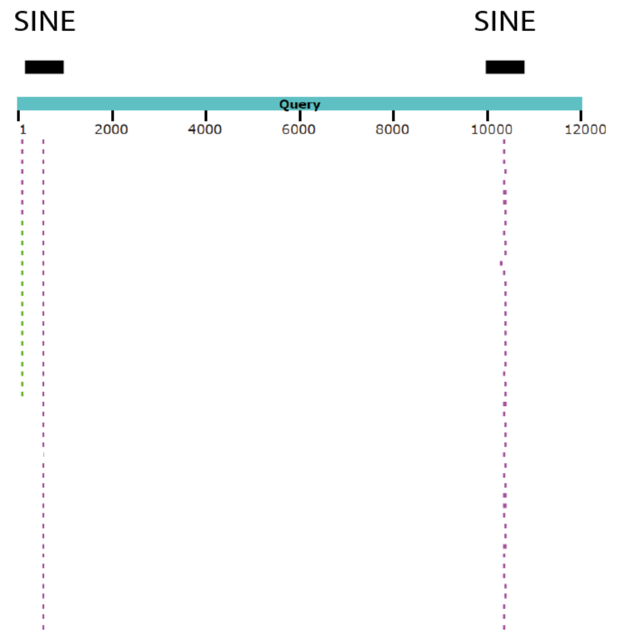

5	14:105058665-105064665	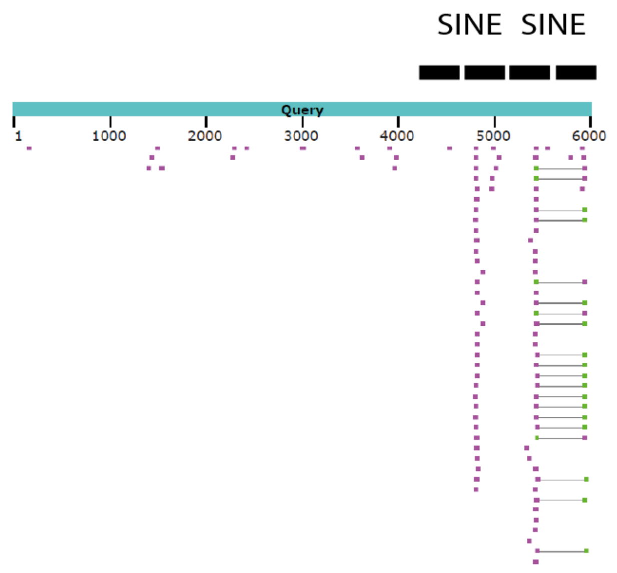
6	14:105069641-105075641	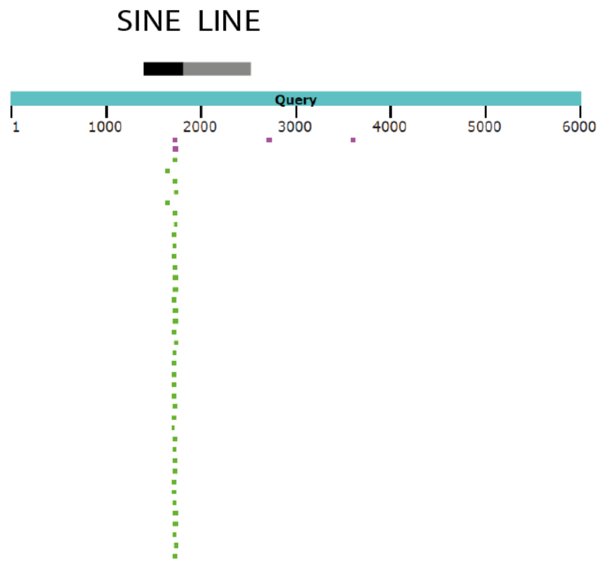
7	14:105555400-105563600	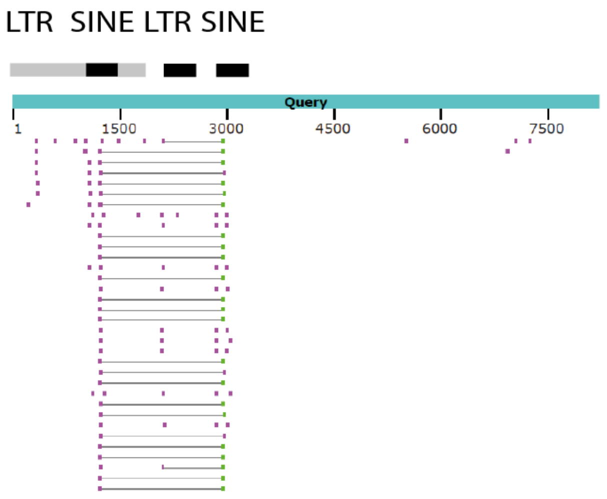

8	14:105579331-105585331	no repeats 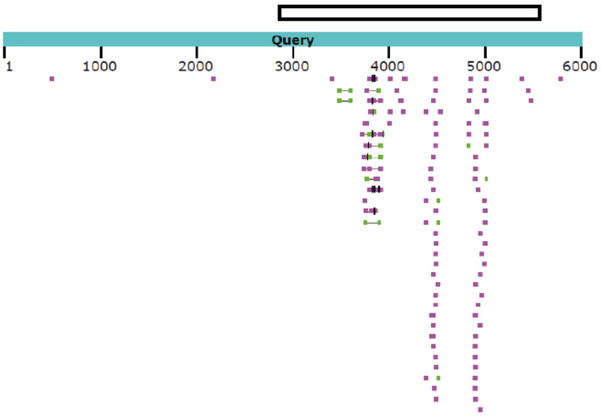
9	14:105628900-105634900	no repeats LTR DNA LTR 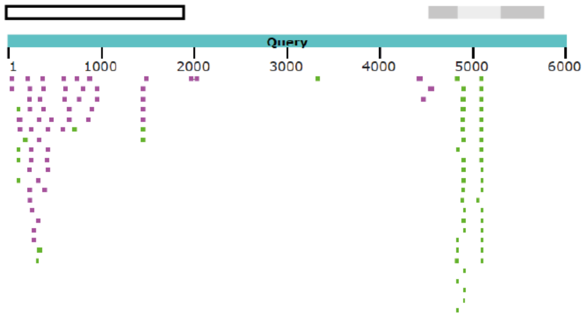
10	14:105674707-105683021	LTR SINE LTR SINE 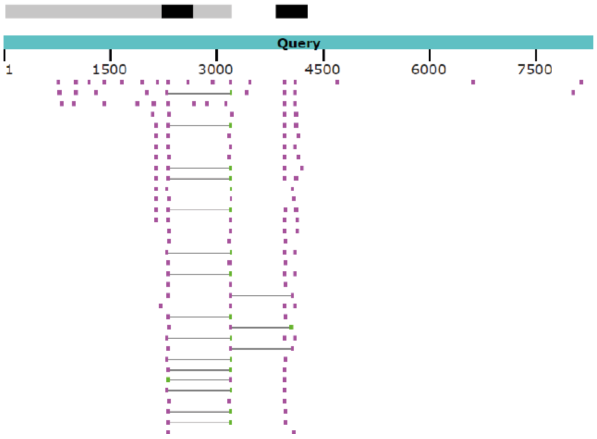

<table>
<tr><td>11</td><td>14:105744600-105750600</td><td>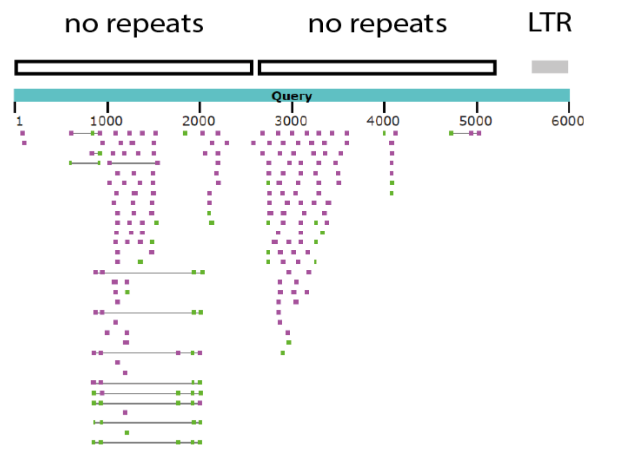
</td></tr>
<tr><td>12</td><td>14:106398526-106404526</td><td>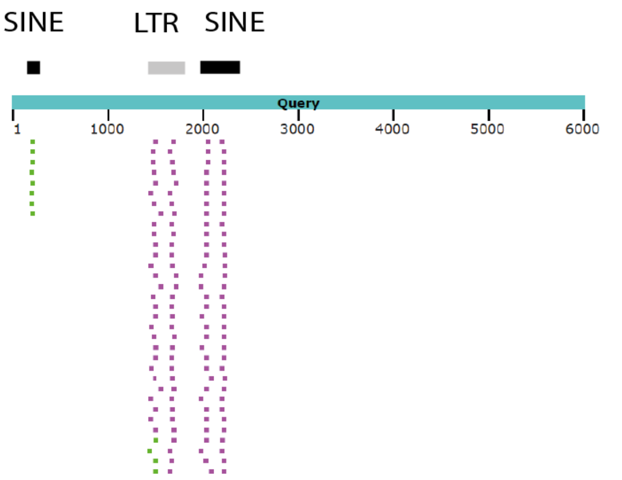
</td></tr>
<tr><td>13</td><td>14:67458397-67464397</td><td>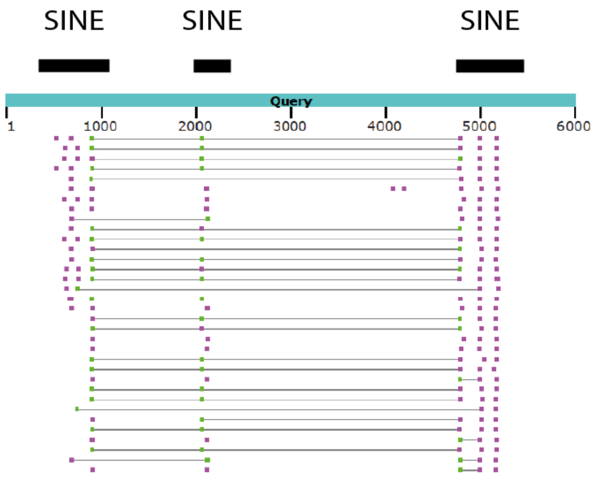
</td></tr>
</table>

14	14:67523837-67529837	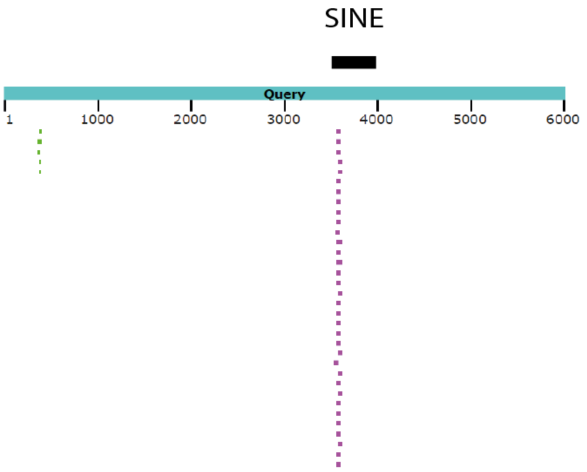
